# Diffusion Imaging of Auditory and Auditory-Limbic Connectivity in Tinnitus: Preliminary Evidence and Methodological Challenges

**DOI:** 10.1155/2014/145943

**Published:** 2014-06-22

**Authors:** Anna Seydell-Greenwald, Erika P. Raven, Amber M. Leaver, Ted K. Turesky, Josef P. Rauschecker

**Affiliations:** ^1^Laboratory of Integrative Neuroscience and Cognition, Department of Neuroscience, Georgetown University Medical Center, 3970 Reservoir Road NW, New Research Building, Washington, DC 20007, USA; ^2^Center for Brain Plasticity and Recovery, Department of Neurology, Georgetown University Medical Center, 4000 Reservoir Road NW, Building D, Washington, DC 20007, USA; ^3^Interdisciplinary Program in Neuroscience, Department of Neurology, Georgetown University Medical Center, 4000 Reservoir Road NW, Building D, Washington, DC 20007, USA; ^4^Ahmanson-Lovelace Brain Mapping Center, Department of Neurology, 635 Charles Young Drive South, Suite 225, Los Angeles, CA 90095, USA; ^5^Interdisciplinary Program in Neuroscience, Department of Neuroscience, Georgetown University Medical Center, 4000 Reservoir Road NW, Building D, Washington, DC 20007, USA

## Abstract

Subjective tinnitus, or “ringing in the ears,” is perceived by 10 to 15 percent of the adult population and causes significant suffering in a subset of patients. While it was originally thought of as a purely auditory phenomenon, there is increasing evidence that the limbic system influences whether and how tinnitus is perceived, far beyond merely determining the patient's emotional reaction to the phantom sound. Based on functional imaging and electrophysiological data, recent articles frame tinnitus as a “network problem” arising from abnormalities in auditory-limbic interactions. Diffusion-weighted magnetic resonance imaging is a noninvasive method for investigating anatomical connections in vivo. It thus has the potential to provide anatomical evidence for the proposed changes in auditory-limbic connectivity. However, the few diffusion imaging studies of tinnitus performed to date have inconsistent results. In the present paper, we briefly summarize the results of previous studies, aiming to reconcile their results. After detailing analysis methods, we then report findings from a new dataset. We conclude that while there is some evidence for tinnitus-related increases in auditory and auditory-limbic connectivity that counteract hearing-loss related decreases in auditory connectivity, these results should be considered preliminary until several technical challenges have been overcome.

## 1. Introduction

Subjective tinnitus, an auditory phantom percept often described as “ringing in the ears,” affects about 10 to 15% of the adult population [1] and significantly impairs quality of life in a subset of those affected by it. While being often perceived “in the ears” and linked to hearing loss in the vast majority of cases, chronic subjective tinnitus appears to be a problem of the central nervous system rather than the ear, since it can persist or even start when the auditory nerve is cut [2]. Numerous studies in human tinnitus patients as well as animal models of tinnitus have provided evidence for structural and functional changes at multiple locations of the central auditory system, and it is widely assumed that central auditory system plasticity is at the root of the aberrant neural activity that gives rise to the perception of tinnitus [3].

However, central auditory system plasticity alone cannot explain the phenomenon of tinnitus. First, compensatory plasticity should occur in all cases of significant deafferentation, yet tinnitus is only reported by a subset of patients with measurable hearing loss [4]. Second, tinnitus patients often report that tinnitus is exacerbated or even triggered by stress [5, 6], which suggests influences of the limbic system. Indeed, there is mounting evidence that limbic system involvement in tinnitus goes beyond merely determining the emotional response to a chronic and sometimes debilitating condition but may instead modulate whether and to what extent aberrant auditory system activity results in a conscious tinnitus percept [7–12]. Of particular interest in this context are previous findings indicating reduced gray matter (GM) in subcallosal prefrontal cortex [10, 11, 13] and the amygdala-hippocampal area [14]; tinnitus-related hyperactivity in the ventral striatum near the nucleus accumbens (NAc) whose strength was correlated with prefrontal GM reductions [10]; correlations between ventromedial prefrontal cortex (vmPFC) function and tinnitus-related variables in ventromedial prefrontal cortex [12, 15]; and modulation of the tinnitus percept by electrical stimulation of the striatum [7].

In line with these findings, many theoretical models frame tinnitus as a network problem, arising from altered interactions between multiple auditory and limbic-related brain structures [10, 16–21]. Consequently, tinnitus research has increasingly employed methods that interrogate large-scale brain networks and interactions between them, in studies of functional connectivity using whole-head magnetoencephalography [22–24], EEG [25–27], and fMRI [28–33]. In addition to investigating tinnitus-related abnormalities in functional connectivity, there is also an increasing interest in assessing potential alterations in anatomical connectivity that might arise from or underlie the observed alterations in functional connectivity and other imaging measures. An increasingly popular tool for assessing structural connectivity in the human brain in vivo is diffusion tensor imaging.

Diffusion-weighted magnetic resonance imaging (DWI) is noninvasive means of measuring water diffusion in tissue. Because water diffusion is hindered by myelin sheaths, axonal cell membranes, and neurofilaments, it is much stronger in the direction parallel to major fiber tracts than in the direction perpendicular to the tracts [34]. By measuring water diffusion along multiple noncollinear directions and fitting a “diffusion tensor” describing diffusion in each direction as well as the correlations between the directions, diffusion tensor imaging (DTI) allows the derivation of measures such as mean diffusivity (MD), fractional anisotropy (FA), and the principal diffusion direction. One can visualize a diffusion tensor as an ellipsoid; the principal axis corresponds to the principal diffusion direction, the average volume corresponds to MD, and the elongation corresponds to FA.

All three measures are used to make inferences about white matter [35]. The principal diffusion direction is interpreted as an estimate of the dominant direction of the fiber tracts, which is then used to track fibers between remote locations in vivo. Fractional anisotropy is commonly used as an indicator of white matter microstructural integrity. The reasoning behind this interpretation is that FA should be the largest in regions where strongly myelinated fiber tracts run in parallel, permitting free diffusion along, but preventing diffusion perpendicular to, the fibers. Thus, reductions in myelination or in the number of parallel fibers result in lower FA. Low FA should be observed when diffusion is equally strong in all directions, for example, where oriented microstructures are essentially absent (such as the ventricles) and in regions with a high density of fibers oriented in many different directions. Because it is a measure of overall diffusion regardless of direction, mean diffusivity can distinguish between these cases: in the former, MD would be high because diffusion would be unconstrained in all directions and in the latter, MD would be low due to the presence of myelinated fibers through which water molecules cannot easily cross.

Note, however, that making these inferences requires several strong assumptions and that the tensor (ellipsoid) model of diffusion is only appropriate if there is only one fiber bundle running in a straight line through the voxel. As pointed out by Jones and colleagues [36], due to the relatively large voxel sizes necessary for maintaining reasonable signal-to-noise ratio (SNR), it can be assumed that at least 90% of voxels contain more than one fiber population, thus violating the core assumption behind the tensor model. Moreover, large voxels are also likely to contain different tissue types (i.e., not only white, but also gray matter) and/or cerebrospinal fluid (CSF), resulting in partial-volume effects. Thus, while DTI measures such as FA and MD are certainly sensitive to changes in tissue microstructure (e.g., axon diameter and density, myelination, and membrane permeability), one should bear in mind that interpreting a difference in these measures with regard to a particular anatomical change (e.g., loss of fibers) or even to a rather vague property such as “white matter integrity” or using them to quantify the strength of anatomical connections between brain areas is a huge leap from the data based on assumptions that in all likelihood are severely violated [36].

Few studies to date have investigated tinnitus using DTI, and their results have been somewhat divergent. The first DTI studies of tinnitus in humans constrained their analyses to predefined regions of interests (ROIs). Lee et al. [37] compared average FA in small, circular ROIs in the corpus callosum, frontal arcuate fasciculus, and parietal arcuate fasciculus between a group of 28 tinnitus patients and 12 normal hearing controls. Average FA was found to be significantly lower in the left frontal arcuate fasciculus and the right parietal arcuate fasciculus. However, both age and hearing loss were higher in the patient group than in the control group. While the authors could mitigate age differences as an alternative explanation for the FA reductions in patients by showing that there were no significant correlations between FA and age in the relevant ROIs, hearing loss differences could not be similarly ruled out. Crippa et al. [38] used DTI-based probabilistic fiber tracking to assess white matter tracts connecting the inferior colliculi (IC), auditory cortices (ACx), and amygdalae (AM) in 15 tinnitus patients and 10 control participants, matched for age. A higher percentage of fibers tracked from ACx reached the ipsilateral AM in tinnitus patients than in controls, and the same held for tracking success from left ACx to left IC and from right IC to right AM. The authors interpret these findings as indicating stronger auditory-limbic connectivity in tinnitus patients.

Three additional DTI studies investigating tinnitus in humans did not constrain their analysis to certain ROIs but instead searched for tinnitus-related connectivity changes along all major white matter tracts. Aldhafeeri et al. [39] found decreased FA in the left longitudinal fasciculus, as well as in the left superior longitudinal fasciculus, the left anterior thalamic radiation, the body and splenium of the corpus callosum, and the right prefrontal cortex of tinnitus patients compared to controls. These FA reductions are reminiscent of those reported by Lee et al. [37]. In that study, it was unclear whether the FA reductions were due to tinnitus or hearing loss. Aldhafeeri et al. [39] report that average hearing thresholds did not differ significantly between tinnitus patients and controls, suggesting that, in their data, FA reductions are indeed due to tinnitus, not hearing loss. However, the only two test frequencies specifically mentioned in the article are 2 kHz and 4 kHz, and it is unclear whether the comparison of hearing thresholds included the higher frequency range that is most commonly affected in tinnitus patients. Thus, differences in high-frequency hearing loss could still have contributed to the observed FA reductions. The remaining two studies [40, 41] took hearing loss into account but used different approaches and had quite different results. Husain et al. [40] used a three-group design comparing participants with tinnitus and hearing loss to participants with hearing loss but no tinnitus and to participants with neither hearing loss nor tinnitus. This study observed only differences due to hearing loss; compared to controls, patients with hearing loss had reduced FA in a right hemisphere cluster including the anterior thalamic radiation, inferior longitudinal fasciculus, and inferior frontooccipital fasciculus. Benson et al. [41] compared two groups of patients with noise-induced hearing loss, differing only in tinnitus status, and found that the tinnitus group had increased FA in several clusters along the left anterior thalamic radiation, as well as in some clusters along the left and right superior longitudinal fasciculi, the left inferior longitudinal fasciculus, and the right interior frontooccipital fasciculus.

Taken together, the results of these studies seem to hint at FA reductions associated with hearing loss (directly demonstrated by Husain et al. [40] and indirectly by Lee et al. [37] and Aldhafeeri et al. [39]) and FA increases and increased fiber tracking success between auditory and frontal/limbic areas in tinnitus patients [38, 41]. However, considering the technical difficulties associated with diffusion imaging analysis in general and probabilistic fiber tracking in particular, the small number of studies, and the even smaller number of studies controlling for age and especially hearing loss, more research is clearly needed. Most studies so far have focused on FA as a measure of tract integrity; however, as argued above, this interpretation is problematic due to the high likelihood of crossing fibers and partial-volume effects in the assessed voxels. We believe that including MD as an additional measure can alleviate at least part of the interpretation problem by assessing general (nondirectional) changes in diffusivity, thus providing a clue as to whether the part of the observed FA changes that reflects changes in tissue microstructure should be attributed to changes along the principal diffusion direction. Moreover, despite the increasing interest in auditory-limbic interactions and the role of the limbic system in tinnitus, none of the diffusion imaging studies to date has considered factors such as depression and anxiety, which are often elevated in tinnitus patients and may contribute to the observed connectivity changes. The present study thus investigates FA and MD while looking at effects of tinnitus unrelated to hearing loss and depression/anxiety.

Based on the results of earlier studies (summarized above), we expected to find hearing-loss related FA decreases and MD increases along auditory pathways (specifically, in the white matter near the inferior colliculi, medial geniculate nuclei, and auditory cortices) and tinnitus-related increases in FA (and decreases in MD) along auditory and auditory-limbic pathways. We assumed that these effects would be most clearly reflected in group differences and also expected correlations between subjective tinnitus ratings and diffusion measures in vmPFC and NAc, which according to our model of tinnitus are the key areas modulating the tinnitus percept [19]. Regarding behavioral measures, we expected (based on our own experience with the population as well as on the literature) that tinnitus patients would be more noise sensitive [42], have higher depression and anxiety scores [43], and have stronger hearing loss [44] than age-matched controls.

## 2. Materials and Methods

### 2.1. Participants

DTI data were acquired from 24 tinnitus patients (TPs) and 19 controls (CTs). Both groups comprised a wide range of ages (TPs: 23–66, mean = 50.13, and sd = 14.64; CTs: 27–67, mean = 48.32, and sd = 12.04), included both men and women (12 women in each group), and included left- and right-handers (2 left-handers among the CTs and 3 among the TPs). For various reasons (see “Quality Control and Preprocessing” below), data from several subjects were excluded from the analyses. The results reported here are based on data from 18 TPs (9 female, 3 left-handed) and 14 CTs (10 female, 1 left-handed). The groups did not differ significantly regarding age (TPs: mean = 44.71 and sd = 11.42; CTs: mean = 46.50 and sd = 13.08; *t* = 0.40, *P* = 0.6888) or regarding the proportion of female and left-handed participants (*χ* ^2^ = 1.50, *P* = 0.2208 for sex; *χ* ^2^ = 0.65, *P* = 0.4190 for handedness).

### 2.2. Behavioral Data Acquisition

All participants underwent audiometry at Georgetown University Hospital's Department of Otolaryngology, assessing pure-tone thresholds for both ears from 200 to 20,000 Hz. However, only thresholds up to 8 kHz could be established in all participants. We thus computed average hearing loss (HL) for all frequencies up to 8 kHz for use as a covariate. In addition, all participants completed the Patient Health Questionnaire (PHQ9 [45]), the Generalized Anxiety Disorder questionnaire (GAD7 [46]), and the Hospital Anxiety and Depression Scale (HADS [47]) to assess symptoms of depression and anxiety. All TPs also completed the Tinnitus Handicap Inventory (THI [48]), and both TPs and CTs completed the Tinnitus Sample Case History Questionnaire (TSCHQ [49]; CTs only completed those items that did not specifically address the participant's tinnitus). Of this latter instrument, the items of particular interest in TPs were “*Describe the loudness of your tinnitus using a scale from 1 to 100 (1 = very faint, 100 = very loud)*” and “*What percent of your total awake time, over the last month, have you been aware of your tinnitus? For example, 100% would indicate that you were aware of your tinnitus all the time, and 25% would indicate that you were aware of your tinnitus *1/4* of the time.*” Of particular interest in both CTs and TPs were the items “*Do you have a problem tolerating sounds because they often seem much too loud? That is, do you often find too loud or hurtful sounds which other people around you find quite comfortable?*” and “*Do sounds cause you pain or physical discomfort?*” the answers to which were provided on a rating scale (a slight deviation from the original TSCHQ) and combined into a single noise sensitivity measure for the purpose of the present analysis.

### 2.3. MRI Data Acquisition

Two diffusion-weighted datasets were acquired in immediate succession for each participant on a 3-Tesla Siemens TIM Trio scanner using a 12-channel birdcage head coil. Each dataset contained five non-diffusion-weighted images (gradient value *b* = 0 s/mm^2^—later referred to as “*b*0”) and 30 diffusion-weighted images (gradient value *b* = 1000 s/mm^2^) in which the gradients were applied in 30 noncollinear directions. The parameters used for the DTI sequence were as follows: repetition time (TR) = 7,700 ms, echo time (TE) = 100 ms, 55 horizontal slices, acquired in interleaved order, and 2.5 × 2.5 × 2.5 mm^3^ resolution. A high-resolution T1-weighted structural scan (MPRAGE, TR = 2,530 ms, TE = 3.5 ms, inversion time = 1,100 ms, flip angle = 7°, 176 sagittal slices, and 1 × 1 × 1 mm^3^ resolution) was also acquired in the same session.

### 2.4. MRI Data Analysis

Data analysis was performed using FSL (version 5.0.0) as provided by the University of Oxford's Center for Functional Magnetic Resonance Imaging of the Brain (FMRIB). Descriptions of the FSL software have been provided in multiple publications [50–52]. At the core of the present analysis were several functions of FMRIB's diffusion toolbox (FDT [53, 54]) which will be described in more detail below.

### 2.5. Quality Control and Preprocessing

The high-resolution T1-weighted scan was inspected to confirm that none of the participants had any large-scale structural abnormalities (e.g., lesions or atrophy unusual for their age). Data from one CT were excluded because of strongly enlarged ventricles.

The two diffusion-weighted datasets acquired from each participant were concatenated in time and visually inspected for excessive motion and artifacts. Based on this inspection, single bad images (displaying an obvious offset between odd and even slices due to in-volume motion) were removed from the datasets of five participants (2 TPs and 3 CTs). Datasets with more than three bad images or excessive motion between subsequent volumes were excluded from the analysis, which removed an additional 8 participants (5 TPs and 3 CTs). Two more datasets were discarded, one (1 CT) because of significant signal loss (“hole artifact”) in superior regions of the brain and one (1 TP) because it was missing the superior-most slices of the brain due to volume placement issues.

Following quality control and exclusion of bad datasets, FSL's “eddycorrect” function was used to automatically align all images acquired for a subject to the first non-diffusion-weighted (*b*0) image of that subject using 12-parameter affine transformation. This function corrects for motion between successive images as well as for image distortions caused by eddy currents, which differ for images acquired with diffusion weighting in different directions. In order to obtain a mask for limiting further analysis steps to voxels inside the brain, FSL's brain extraction tool (BET [55]) was used on the first *b*0 image of each subject. Parameters were adjusted and manual corrections were made as necessary to ensure that the resulting mask included all brain tissue while excluding most or all of the surrounding skull and meninges.

### 2.6. Tensor Fitting

Following the above preprocessing, a diffusion tensor was estimated for each voxel in each subject's concatenated dataset using FSL's “dtifit” function. Aside from the 4D dataset and the mask file constraining the analysis to voxels in the brain, this function also takes as input two text files, one describing the gradient directions with which each of the images in the 4D dataset was acquired (the bvecs file) and one describing the diffusion weighting applied when acquiring each image (*b* values, which in the present dataset were 0 s/mm^2^ for non-diffusion-weighted images and 1000 s/mm^2^ for diffusion-weighted images). Like the two 4D datasets acquired for each subject, these files, too, were concatenated, and where single images had been removed from the 4D files during the quality control process, the corresponding entries were removed from the bvecs and bvals files.

After tensor fitting, the resulting functional anisotropy (FA) and mean diffusivity (MD) maps were inspected, separately, for each subject. All maps looked as expected (i.e., higher FA values in locations of major white matter tracts, such as the corpus callosum, and higher MD in locations of cerebrospinal fluid, such as the ventricles), and no artifacts were found.

### 2.7. Preparation for Tract-Based Spatial Statistics (TBSS)

Whole-brain analyses aiming to compare groups of subjects require that all subjects' 3D datasets be aligned in a shared standard space (such as Talairach or MNI space). Because of individual differences in brain anatomy, perfect alignment cannot be achieved by linear affine transformations, and alignment procedures allowing for local distortions (i.e., nonlinear alignment) run the risk of resulting in perfectly aligned datasets that no longer reflect the original data well. This is particularly problematic in regions of high individual variability. Residual misalignments between subjects can, to a certain degree, be overcome by large-scale smoothing of the data (which “blurs away” individual differences), but large amounts of smoothing also introduce partial-volume effects and can conceal small, spatially circumscribed effects. We thus decided to use a method that avoids excessive smoothing.

The tract-based spatial statistics (TBSS) approach [56] to analyzing multisubject diffusion data overcomes these problems in two ways. First, it constrains the analysis to the main white matter tracts that can be assumed to be present and laid out similarly in all subjects. Second, rather than aiming to transform all subjects' diffusion data such that their main white matter tracts are perfectly aligned, it only roughly aligns all subjects' data, thus avoiding excessive distortion of individual datasets. This results in a multisubject dataset in which the mean white matter tracts are not perfectly aligned but sufficiently well aligned to allow derivation of an average white matter (WM) skeleton containing only the main tracts shared across subjects. After eroding the average skeleton such that only the centers of the main tracts are left, individual datasets are searched in the direction perpendicular to the average tract until the center (i.e., the maximum FA value) of the corresponding individual tract is found. The data from the individual tract center are then projected onto the average WM skeleton. This ensures that subsequent statistical analyses compare corresponding points of all individuals' white matter tract centers.

In the present analysis, we prepared for TBSS by using nonlinear transformations to roughly align all subjects' FA data with the FMRIB58 FA template, an average FA image based on the datasets of 58 healthy subjects aged between 20 and 50 years transformed into MNI space. An average FA image was then created from the aligned data and thresholded at FA >0.35 to ensure that only voxels with reasonably high likelihoods of containing white matter tracts in most subjects were retained. (We chose an FA threshold stricter than the recommended range of 0.2 to 0.3 [56] because visual inspection of the average FA image indicated that, at more lenient thresholds, the skeleton included small peripheral WM tracts for which intersubject correspondence cannot safely be assumed.) The resulting image was then eroded such that only the centers of the white matter tracts (i.e., the voxels with highest FA values) remained. Data from each individual subject's tract center were then projected onto this average WM skeleton as described above.

### 2.8. Group Comparisons across the Entire WM Skeleton

Data were analyzed using an analysis of covariance (ANCOVA) approach, investigating group differences in FA and MD while controlling for the effects of age and hearing loss (two variables known to affect connectivity from many previous studies), using a design matrix including four predictors: two binary predictors coding group membership (CT and TP) and two continuous predictors coding age and average hearing loss, respectively. The hearing loss predictor was orthogonalized with respect to age to account for the known positive correlation between age and hearing loss.

Across the entire WM skeleton, we tested both FA and MD values for group differences, as well as correlations with age and hearing loss, using FSL's “randomise” tool with 10,000 iterations and threshold-free cluster enhancement (TFCE [57]). The advantage of the TFCE approach is that, unlike other cluster-size- (or cluster-mass-) based approaches, it does not require the user to arbitrarily define a cluster-forming threshold (i.e., a threshold that voxels have to exceed in order to be counted as part of a cluster whose size or mass is then evaluated for significance by testing it against a null distribution obtained via permutation testing). Instead, for each voxel, it essentially uses all possible cluster-forming thresholds from 0 up to the statistical value of the voxel, establishing the cluster extent at each of these thresholds, and then summarizes the results as a weighted sum of all extents at all thresholds (for more details, see [57]). The resulting statistical image retains important spatial features of the original statistical image, such as local maxima, while at the same time enhancing each voxel's signal depending on how much “support” it receives from neighboring voxels with high statistical values. Like the original statistical image, the TFCE image can be converted into a map of *P* values corrected for multiple comparisons using nonparametric permutation testing, as implemented in FLS's randomise function. Because the WM skeleton is only two-dimensional at any given point in space, we used the “−T2” option of the FSL randomise tool, which is optimized for 2D data.

### 2.9. Group Comparisons across Auditory and Limbic Regions of Interest

To increase power for detecting smaller effects in locations where such effects were expected, we repeated our search for group differences while constraining single-voxel analyses to one region of interest (ROI) at a time. Twelve auditory and limbic ROIs were defined based on our theoretical framework and previous findings: left and right auditory cortices (ACx), medial geniculate nuclei (MGN), inferior colliculi (IC), amygdalae (AM), accumbens nuclei (NAc), and ventromedial prefrontal cortex (vmPFC) white matter.

**Figure 1:**
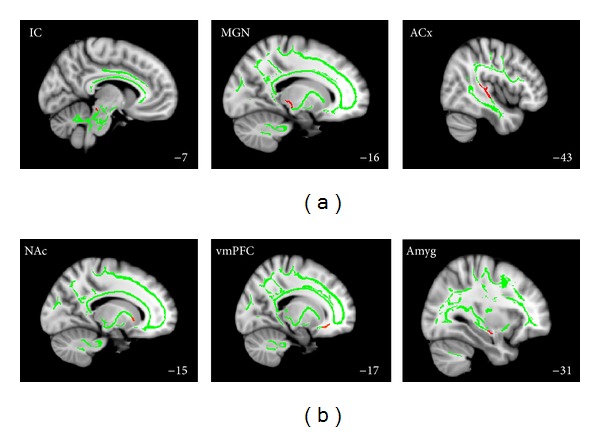
*Regions of interest.* Auditory (a) and limbic (b) ROIs (red) overlayed on the WM skeleton (green) superimposed on the MNI152 brain template. Numbers in the bottom right corner indicate the MNI *x*-coordinate of the illustrated sagittal slice.

Left and right ACx ROIs were defined as all voxels on the average WM skeleton of Heschl's gyrus (HG) and the planum temporale (PT) that were anterior to *y* = −36. Medial geniculate nucleus ROIs were defined as all WM skeleton voxels falling within a sphere of an 8 mm radius around MNI coordinates +/−17, −24, −2 (following Mühlau et al. [13]), and ROIs for the inferior colliculi (IC) were defined as all WM skeleton voxels falling between the MNI coordinates of *x* = +/−3 to +/−8, *y* = −30 to −35, and *z* = −9 to −17, that is, as the WM tracts inferior to the IC (since the IC themselves were not part of the WM skeleton). Amygdala ROIs were defined as all voxels on the WM skeleton falling within the area defined by the Harvard-Oxford Subcortical Structures Atlas as having a nonzero probability of belonging to the amygdala (the extremely lenient lower bound was chosen so that nearby white matter would be included) while at the same time falling within the WM tracts identified by Crippa et al. [38] as connecting the amygdala with the auditory system. The white matter tracts of the anterior limb of the internal capsule nearest to the left and right NAc were defined as our NAc ROIs, and inferior frontal WM tracts extending from just anterior to the head of the caudate into the area inferior to the genu of the corpus callosum were defined as our vmPFC ROIs. Details about the ROIs can be found in Table 1 and illustrations can be found in Figure 1.

### 2.10. Analyses for Correlations between Tinnitus Loudness and DTI Measures

In addition to testing for group differences, we also tested average FA and MD for correlations with tinnitus loudness ratings. Tinnitus loudness ratings were chosen as the tinnitus-related variable of interest because they, in contrast to THI scores and tinnitus awareness ratings, did not show strong correlations with depression and anxiety scores (see results for “Behavioral Data” below) and thus appeared to be the “purest” measure of the tinnitus percept itself (as opposed to tinnitus-related distress). We limited our search for correlations to only one tinnitus-related variable of interest to avoid the strict error-level adjustment that would be required if we tested multiple correlations.

We also performed post hoc correlation analyses across voxels displaying a significant group difference or tinnitus-loudness correlation to investigate whether the observed effects might be due to any of the other variables (e.g., depression, anxiety, and noise sensitivity). (We chose this post hoc approach rather than including these variables as covariates of no interest because inspection of the scores made it obvious that the homogeneity of regression slopes assumption was not met for these variables, making them unfit for inclusion as covariates.) While this involves multiple tests, we nevertheless used an uncorrected error level as our significance criterion, reasoning that since we were trying to demonstrate the absence of correlations, using a more lenient threshold would make the test stricter. At an uncorrected significance threshold of *P* < 0.05, correlations across the 18 TPs would have to exceed an absolute value of 0.469, correlations across the 14 CTs an absolute value of 0.532, and correlations across all 32 participants an absolute value of 0.347 to be considered significant.

## 3. Results

### 3.1. Behavioral Data

While matched for age, the two participant groups nevertheless differed significantly regarding auditory behavioral measures. Averaged across all frequencies of the standard audiogram (i.e., up to 8 kHz), TPs had significantly more hearing loss than CTs (27.14 dB HL versus 13.60 dB HL; *P* = 0.0013). In addition, TPs indicated significantly higher sensitivity to noise on the TSCHQ (*P* = 0.0005). The groups did not differ regarding depression and anxiety measures, although there were strong tendencies for TPs to score higher on the associated questionnaires (*P* = 0.1572 for the PHQ9 and *P* = 0.0506 for the GAD7). In addition, both tinnitus awareness ratings and THI scores were strongly correlated with depression and anxiety scores (correlations ranging from 0.53 to 0.78). In contrast, tinnitus loudness ratings were comparatively unrelated to depression and anxiety measures (all correlations below 0.25).

### 3.2. Whole-Skeleton Analysis regarding FA and MD

Our first DTI analysis tested all voxels of the average WM skeleton for significant group differences and correlations with age and hearing loss, using the ANCOVA TFCE approach described above. While no significant group differences emerged, there were significant correlations between DTI measures and both age and hearing loss across groups.

### 3.3. FA Decreases and MD Increases with Age

**Figure 2:**
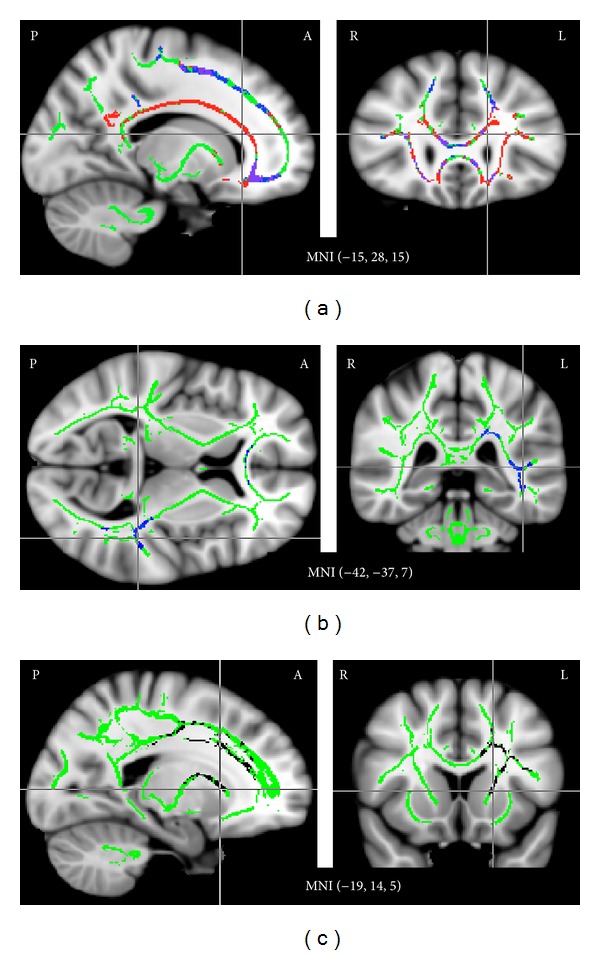
*Correlations between FA/MD and age (a), hearing loss (b), and tinnitus loudness (c)*. (a) Many voxels of the average WM skeleton (green), especially in frontal cortex and the corpus callosum, showed a significant (*P* < 0.05, corrected) positive correlation between age and MD (red), a significant negative correlation between age and FA (blue), or both (purple). (b) In addition, several voxels in the WM tracts near left auditory cortex and in the WM tracts connecting left ACx to the corpus callosum showed a significant negative correlation between average hearing loss and FA (blue). This negative correlation was also observed in anterior portions of the corpus callosum. (c) Significant negative correlations (black) were also observed between tinnitus loudness ratings and MD in the anterior thalamic radiation and the anterior and superior left corona radiata. Results are superimposed on the MNI152 brain template.

Across the entire skeleton, but especially in the frontal WM tracts and the corpus callosum (see Figure 2(a)) and mostly sparing subcortical WM tracts and brainstem, we observed significant positive correlations between MD and age and, more constrained to frontal WM tracts, corresponding negative correlations between FA and age. These age-related decreases in FA and increases in MD (regardless of tinnitus status) are well in line with the results of other DTI studies that specifically investigated the effect of age on white matter tracts (for a recent review, see [58]). No significant effects in the opposite direction (i.e., positive correlations between age and FA and negative correlations between age and MD) were observed. These findings serve as a “sanity check” of sorts, indicating that well-known and robust effects are replicated in our dataset.

### 3.4. FA Decreases with Hearing Loss

More interestingly, we also observed significant negative correlations between FA and average hearing loss in the WM tracts near left auditory cortex and in the WM tracts between left auditory cortex and the corpus callosum (Figure 2(b)). Corresponding effects in right auditory cortex could be seen at slightly reduced thresholds (*P*_corr_ < 0.1). Positive correlations between MD and average hearing loss were evident in the same locations at more lenient thresholds but did not reach significance. In addition, significant negative correlations between FA and average hearing loss were also observed in several voxels of the corpus callosum. Because the variable “average hearing loss” was orthogonalized with respect to age in our analysis, these effects are unlikely to be related to the known age-related decline in FA.

### 3.5. Negative Correlations between MD and Tinnitus Loudness Ratings in Left Anterior Thalamic Radiation and Anterior/Superior Corona Radiata

Significant negative correlations between MD and tinnitus loudness ratings were observed in the anterior thalamic radiation and the anterior and superior corona radiata of the left hemisphere (Figure 2(c)). Corresponding trends (at *P*_corr_ < 0.1) were also evident in the right hemisphere. Interestingly, these effects were found in locations corresponding well to those in which Benson et al. [41] observed higher FA in tinnitus patients than in controls; however, the present dataset did not show any FA effects in these areas.

To investigate whether the observed correlation might be driven by variables other than tinnitus loudness, we extracted average MD across all voxels identified as significant in this correlation analysis (i.e., defining a post hoc ROI) and computed correlations with the remaining tinnitus-related variables. (Note that this post hoc analysis is not statistically independent since the ROI was chosen for correlation between MD and tinnitus loudness and is now being tested for correlations between MD and other tinnitus variables, some of which are correlated with tinnitus loudness.) The negative correlation between MD and tinnitus loudness ratings was corroborated by similarly strong negative correlations between MD and tinnitus awareness (*r* = −0.64, *P* = 0.004) and between MD and noise sensitivity (*r* = −0.60, *P* = 0.008) in TPs; in contrast, CTs did not show a substantial correlation between MD and noise sensitivity (*r* = −0.27, *P* = 0.31). None of the other tinnitus-related variables (hearing loss and depression/anxiety scores) were correlated with MD, neither across nor within groups. In line with the widespread age-related MD increases described above, there was a strong positive correlation (*r* = 0.59, *P* = 0.0004 across groups) between MD and age in this ROI.

### 3.6. ROI Analyses regarding FA and MD

Next, we constrained our search for group differences to certain auditory and limbic ROIs in which we expected significant effects based on our theoretical framework and the results of previous studies. The following paragraphs highlight all significant results (and, where applicable, nonsignificant trends in the contralateral hemisphere); ROIs not mentioned here (MGN, NAc, and AM) did not show significant effects.

### 3.7. FA Increases and MD Reductions in ACx WM

**Figure 3:**
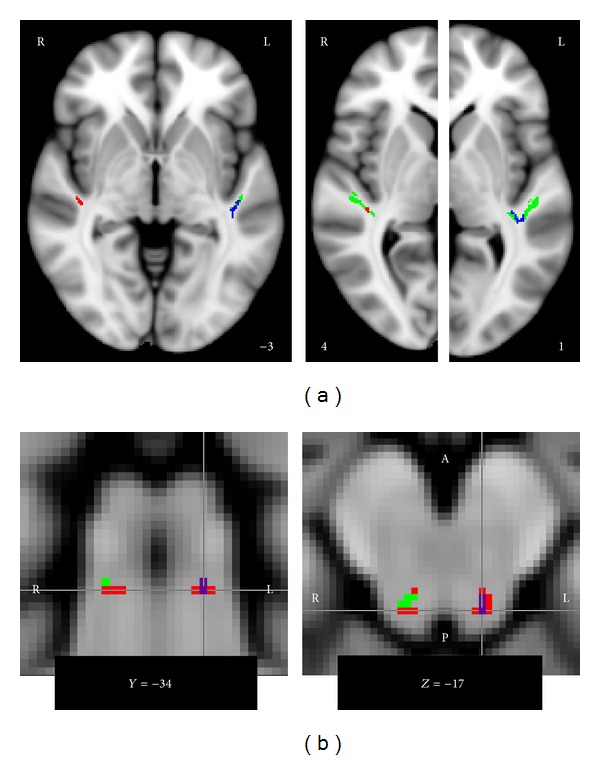
*Group differences regarding FA and MD in auditory ROIs.* (a) Compared to controls, tinnitus patients showed significantly increased FA (red) in right ACx and significantly decreased MD (blue) in left ACx, with corresponding but nonsignificant trends in the opposite hemisphere. The ROIs are indicated in green, and the numbers at the lower edges of the images indicate the MNI *z*-coordinate of the illustrated horizontal slice. (b) Tinnitus patients also showed significantly increased FA (red) in the WM underneath left and right IC and significantly decreased MD (blue) in the WM underneath left IC; a corresponding trend was also present in right IC. Voxels for which both FA increases and MD increases were significant are shown in purple. The numbers at the lower edges of the images indicate the MNI coordinate of the illustrated coronal/horizontal slice.

As shown in Figure 3(a), several voxels of our rACx ROI at the confluence of Heschl's gyrus (HG) and the superior temporal gyrus (STG) had significantly higher FA values in TPs than in CTs (cluster center of gravity (CoG) = 41, −28, 4), and a similar trend was evident (at *P*_corr_ < 0.1) in lACx (CoG = −39, −32, 2). For MD, opposite results were observed: significantly lower MD values in TPs than in CTs in lACx (CoG = −42, −28, −1) and a nonsignificant trend in rACx (CoG = 40, −28, 3). To ensure that the observed effects were not simply due to correcting for hearing loss (which was higher in TPs and, as we mentioned above, associated with lower FA and higher MD), we also repeated the analysis without covariates and found somewhat reduced effects in the same direction, with only the MD effect in lACx remaining significant, the FA effect in lACx trending at *P*_corr_ < 0.1, and the effects in rACx trending at *P*_corr_ < 0.2.

To test whether the observed group differences might be due to noise sensitivity, which was significantly higher in TPs but was not included as a covariate of no interest in the group analysis, we also computed correlations between noise sensitivity and the DTI measure showing the group difference, averaged across all voxels for which the group difference was significant. Across groups and within CTs, these correlations were close to zero. In rACx only, TPs showed a strong negative correlation (*r* = −0.67) between FA and noise sensitivity. Interestingly, this correlation is in opposition to the group difference. As a group, TPs had higher FA and higher noise sensitivity, but within TPs, FA decreased with increasing noise sensitivity so that the TPs with the highest noise sensitivity ratings had FA values more like CTs. Considering these findings, it is highly unlikely that group differences in noise sensitivity were responsible for the observed group difference in the DTI measures. The strong negative correlation between TPs' noise sensitivity and FA in rACx is somewhat puzzling; however, since no corresponding correlation was evident in lACx (*r* = −0.08) or in CTs (*r* = 0.07), it is likely a spurious result.

### 3.8. FA Increases and MD Reductions in the WM Inferior to the IC

We also observed significantly increased FA values and decreased MD values for TPs compared to CTs in the WM inferior to our lIC ROI and significantly increased FA values in rIC (Figure 3(b)). A trend for decreased MD in the rIC ROI was also present (at *P*_corr_ < 0.1). As above, we confirmed that this effect also held when age and hearing loss were not included as covariates. We also checked for correlations between FA/MD (averaged across all voxels of the ROI for which the group difference was significant) and noise sensitivity and found the correlations to be near zero across groups and tending to oppose the group difference when looking at the groups separately, making it extremely unlikely that the observed DTI results reflect differences in noise sensitivity.

### 3.9. The Louder the Tinnitus, the Higher FA and the Lower MD in vmPFC

**Figure 4:**
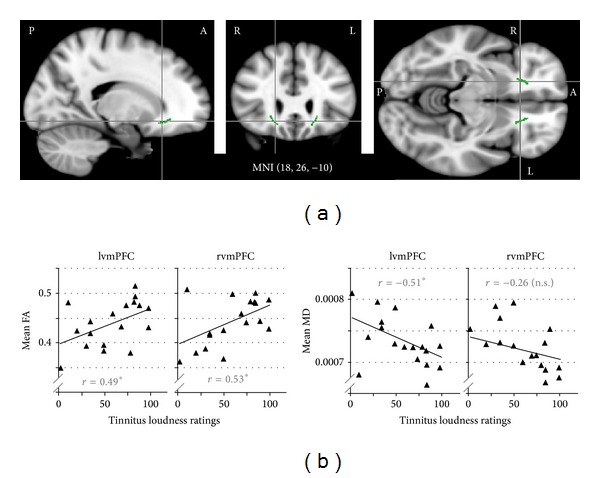
*Correlations between DTI measures (FA and MD) and tinnitus loudness ratings in vmPFC.* (a) Sagittal, coronal, and horizontal views of the anatomically defined vmPFC ROIs (green) superimposed on the MNI 152 brain template. MNI coordinates indicate the crosshairs intersection. (b) Scatter plots illustrating correlations between DTI measures and tinnitus loudness ratings in both ROIs.

In addition to ruling out correlations between DTI measures and noise sensitivity in regions showing significant DTI group differences, we also tested all ROIs for correlations between FA/MD (averaged across all voxels of the ROI) and our tinnitus variable of interest: tinnitus loudness ratings. The only ROIs in which these correlations exceeded the significance criterion (absolute correlation value of 0.47 or larger) were left and right vmPFC. Both showed significant positive correlations between FA and tinnitus loudness ratings (*r* = 0.49, *P* = 0.039 and *r* = 0.53, *P* = 0.024 for left and right vmPFC, resp.); in addition, left vmPFC also showed a corresponding negative correlation between MD and tinnitus loudness ratings (*r* = −0.51, *P* = 0.031). A trend in the same direction (*r* = −0.26, *P* = 0.297) was also evident in right vmPFC. The vmPFC ROIs and scatter plots illustrating the correlations are shown in Figure 4.

These correlations between DTI measures and tinnitus loudness ratings opposed the ones observed in the same ROIs for age and HL: FA decreased and MD increased with age and hearing loss both across and within groups. Correlations with noise sensitivity went in the same direction as those with tinnitus loudness ratings but were weaker and did not reach significance even if an uncorrected criterion was applied or did any of the correlations with depression and anxiety scores. Thus, we are confident that the observed correlations were indeed related to tinnitus rather than to age, hearing loss, noise sensitivity, or depression and anxiety. The correlations were further supported by nonsignificant trends regarding group differences: FA tended to be higher and MD tended to be lower in TPs compared to CTs when correcting for the influence of age and hearing loss.

## 4. Discussion

### 4.1. Summary of Results

In the present dataset, we observed (1) that age was positively correlated with mean diffusivity (MD) and negatively correlated with fractional anisotropy (FA), especially in frontal white matter tracts; (2) that FA was negatively correlated and MD tended to positively correlate with hearing loss in the white matter (WM) between left auditory cortex and the corpus callosum and including both structures; (3) that tinnitus loudness ratings were negatively correlated with MD in the anterior thalamic radiation and anterior and superior corona radiata (although significantly so only in the left hemisphere); (4) that compared to age-matched controls, tinnitus patients had higher FA and lower MD in anatomically defined regions of interest in the white matter tracts underneath both auditory cortices (ACx) and inferior colliculi (IC); and (5) that in anatomically defined ROIs in ventromedial prefrontal cortex (vmPFC), FA correlated positively and MD correlated negatively with tinnitus loudness ratings. Depression and anxiety, while tending to be higher in tinnitus patients than in controls, could be ruled out as alternative explanations for these findings.

Bearing in mind that making inferences from FA/MD findings about microstructural changes is problematic for the reasons outlined in the Introduction section and summarized by Jones and colleagues [36], the age-related findings may be cautiously interpreted as a widespread decline in white matter tract integrity with age. This conclusion has been drawn from a number of studies investigating aging with diffusion-weighted imaging [58]. The present study found hearing loss to be associated with an additional decline in the white matter tracts of the auditory cortices. Interestingly, the presence of a tinnitus percept seemed to more than compensate for this hearing-loss related decrease, since tinnitus patients, despite having significantly more hearing loss than controls, showed an FA/MD pattern commonly interpreted as indicating increased tract density in auditory ROIs. The fact that the same pattern (increased FA and reduced MD) was found to correlate with tinnitus loudness ratings within frontal white matter, especially in the vmPFC ROIs, provides additional evidence for a role of these limbic-related areas in tinnitus.

### 4.2. Comparison with Other DTI Studies of Tinnitus

The present results both confirm and complement those of earlier studies. Hearing-loss related reductions in WM tract integrity have been inferred from several studies for a number of major WM tracts [40, 59] as well as for subcortical auditory ROIs [60–62]; however, the only study [59] reporting such effects in the white matter tracts of Heschl's gyrus and the superior temporal gyrus (i.e., in auditory cortical white matter) was based on a group comparison of young adults without hearing loss and older adults with hearing loss, so that the results might have been due to age rather than hearing loss. Thus, the present finding of a correlation between hearing loss and reduced FA/increased MD in auditory cortical white matter after controlling for age (Figure 2(b)) adds to these previous findings. What none of the DTI studies to date can determine is whether the apparent WM tract reductions are the cause or the consequence of hearing loss. They might reflect the effects of peripheral hearing loss on the central auditory system, that is, a degeneration of connections used less due to reduced input. Alternatively, they may be at the heart of central hearing loss, where an auditory signal is taken up in the periphery but insufficiently propagated through the central auditory system. Lastly, both relationships may hold to some extent, even within the same patient, and contribute to hearing problems.

Our observation of increased FA (Figure 3(b)) near the inferior colliculi of human tinnitus patients compared to controls adds to previous findings of FA increases in subcortical auditory structures (IC and MGN) in a rat model of blast-induced tinnitus [63]. Interestingly, Lutz et al. [59] also reported FA increases in the inferior colliculi for a comparison of older participants with hearing loss with a young normal-hearing control group. In contrast, Lin et al. [61] reported hearing-loss related FA decreases, and our study did not find effects of either age or hearing loss on FA in the inferior colliculi but did find increased FA in tinnitus patients. Considering the high incidence of tinnitus among older participants with hearing loss (which makes it likely that at least some of the older participant in Lutz et al.'s study had tinnitus), this may suggest that, rather than reflecting normal aging of the acoustic pathway (as Lutz et al. [59] conclude), the observed FA increases may instead be a sign of excessive compensatory plasticity following hearing loss that results in tinnitus.

The present results also indicate tinnitus-related FA increases/MD decreases in auditory cortical WM (Figure 3(a)). This is well in line with the increased tracking success in tinnitus patients compared to controls for fibers leaving auditory cortex in the direction of the amygdala and in the direction of the inferior colliculi as reported by Crippa et al. [38]. While it may appear puzzling that no more diffusion studies have observed effects in auditory cortical WM, this is actually not surprising for the following reasons. First, of the five studies investigating tinnitus with diffusion imaging, only four included this region (Lee et al. [37] instead focused on small ROIs in major WM tracts). Of those four, one [39] used an intersubject alignment approach that is bound to fail in auditory cortex, where anatomy can differ vastly between subjects. Two others [40, 41] overcame these alignment issues by using the tract-based spatial statistics (TBSS) approach described above, but neither used threshold-free cluster enhancement (TFCE), which may have led them to miss spatially small effects that did not meet their cluster-size threshold. Also, in the TBSS approach, how much auditory cortical WM is actually included in the average WM skeleton critically depends on the number of participants (which was relatively small in Husain et al.'s study) and on the FA threshold chosen to limit the analysis to major WM tracts; it is thus unclear how much of auditory cortex was included in these previous analyses. In the only two studies that did detect auditory cortex effects (Crippa et al. [38] and the present study), auditory cortex was specifically chosen as an ROI. This suggests that while tinnitus-related changes in this area are present, they may not be easily detected with a spatially rather coarse technique like diffusion imaging. A possible reason for this is the potentially large heterogeneity across study participants regarding auditory experience aside from tinnitus and hearing loss (e.g., musical training) that can also influence auditory cortex connectivity (e.g., [64]).

Lastly, our finding of positive correlations between tinnitus loudness ratings and DTI measures in the vmPFC ROIs (Figure 4), the anterior thalamic radiation, and the anterior and superior corona radiata (Figure 2(c)) and the fact that these effects were stronger in the left than in the right hemisphere fits well with the left-dominant FA increases in frontal and thalamic white matter recently reported by Benson et al. [41] for a comparison of tinnitus patients to controls matched for age and hearing loss. We did not observe such group differences in our own data, but that may be due to the fact that our groups were much more heterogeneous than those studied by Benson et al. [41], whose inclusion criteria required noise-induced hearing loss and, for tinnitus patients, a THI score of at least 35. However, allowing for a wide range of hearing loss and tinnitus severity in our sample enabled us to find correlations that would otherwise have been missed and that nicely complement the group differences observed in previous studies. 

### 4.3. Interpretation

#### 4.3.1. Increased Connectivity within the Auditory System

The number of studies investigating anatomical connectivity in tinnitus by means of diffusion imaging is still relatively small, and no single study so far has given a conclusive picture. This is partly owed to technical difficulties of diffusion imaging (e.g., intersubject alignment issues and limited success of fiber tracking attempts) and partly to the fact that it is nearly impossible to control for and/or investigate the influence of the many potential confounding variables (such as hearing loss, noise sensitivity, and depression/anxiety) at once. Nevertheless, taken together, the evidence is solidifying to suggest that, while hearing loss is related to changes in diffusion measures commonly taken to indicate decreases in white matter integrity within the auditory system at both the subcortical and the cortical level, there is a converse relationship in tinnitus, which is associated with increases in white matter density.

A possible interpretation is that the reduced auditory input associated with hearing loss of peripheral origin results in a weakening of the connections previously carrying this signal. In contrast, propagation of a constant tinnitus signal (arising, e.g., from an increase in spontaneous firing rates of neurons deafferented by hearing loss, as has been observed in several studies and proposed as a potential mechanism for tinnitus generation—e.g., [65]) might lead to preservation or even strengthening of connections. However, since results so far are purely correlational and thus cannot speak to causality, it is also possible that tinnitus is the consequence, rather than the cause, of the observed increase in auditory system connectivity. For example, several studies have found tinnitus to be associated with reorganization of tonotopic maps in auditory cortex. Frequency regions that have lost their normal inputs due to peripheral hearing loss start responding to the same stimuli as adjacent regions, resulting in an overrepresentation of certain frequencies that may be at the heart of the tinnitus signal (e.g., [66, 67]). Such local map reorganization is bound to involve strengthening of local connectivity, although it is unclear whether this effect could be detected given the current resolution of diffusion tensor imaging, where voxels are often several cubic millimeters large. Lastly, it is also possible that both interpretations are true at different locations within the auditory system. Increases in local connectivity may drive the generation of the tinnitus signal, which, being passed on to more remote regions, might in turn drive increased long-range connectivity.

#### 4.3.2. Involvement of the Limbic System

All of the four studies using diffusion imaging in humans and reporting effects that the authors associate with tinnitus [37–39, 41] interpret at least part of their results in terms of altered limbic and/or auditory-limbic connectivity. However, none of these studies attempted to differentiate whether the observed effects were associated with the tinnitus percept per se or rather with its concomitant emotional phenomena. The present study revealed that diffusion measures in vmPFC, as well as along WM tracts containing fibers connecting temporal and thalamic with prefrontal regions, were strongly correlated with tinnitus loudness ratings but not with measures of depression, anxiety, or tinnitus distress. Thus, it provides the first diffusion-imaging evidence for a role of prefrontal, limbic-related areas in determining the tinnitus percept, that is, its intensity, itself.

This evidence adds to previous findings indicating (1) gray-matter reductions in subcallosal prefrontal cortex of tinnitus patients [10, 11, 13] whose magnitude correlates with tinnitus-related hyperactivity in the ventral striatum [10]; (2) correlations between vmPFC activation and tinnitus-related variables [12, 15]; (3) modulatory effects of deep-brain stimulation in the striatum on tinnitus percept [7]. Based on these findings, we have previously proposed a “noise cancellation” model of tinnitus according to which limbic and prefrontal areas work together to evaluate the tinnitus signal and, depending on the relevance assigned to it, enhance or suppress it via feedback to the auditory system. Gray-matter reductions in vmPFC result in a reduced ability to cancel “noise” and are associated with tinnitus-related hyperactivity in ventral striatum and auditory cortex. By demonstrating what might be interpreted as a tinnitus-related increase in auditory-limbic connectivity, diffusion imaging studies like the present one quite literally provide the “missing link” in this model.

In this context, it is interesting to note that the present study observed tinnitus loudness correlations, but no group differences in vmPFC and along some of the same white matter tracts for which Benson et al. [41] reported larger functional anisotropy in tinnitus patients compared to controls. A potential explanation for this discrepancy is that Benson et al. only included tinnitus patients with THI scores of 35 or higher, whereas the majority (14 out of 18) of tinnitus patients included in the present study had THI scores below 35. As mentioned at the beginning of the results section, THI scores in the present study were strongly correlated with depression and anxiety scores (correlations between 0.60 and 0.78). If the same relationship held in Benson et al.'s [41] sample, their patient group was not only more bothered by their tinnitus than ours, but also considerably more depressed and anxious. It is conceivable that increased depression/anxiety results in larger relevance being assigned to the tinnitus signal, leading to its enhancement in a sort of self-perpetuating loop, which goes along with increased limbic-auditory connectivity. The same mechanism could be responsible for the enhanced tracking success between auditory cortex and amygdala reported by Crippa et al. [38]. In other words, the widespread group differences described by Benson et al. [41] and the increased tracking success between auditory cortex and amygdala described by Crippa et al. [38] may be less associated with the tinnitus percept itself and more with the patients' emotional sequelae. These differences would not have shown up in our study because our patients overall had comparatively low depression and anxiety scores. In contrast, connectivity within the circumscribed areas identified in the present study (particularly vmPFC) may modulate the tinnitus percept even in the absence of depression and anxiety and prior to any additional widespread increases in auditory-limbic connectivity that may result from a distressed, self-perpetuating response to it.

### 4.4. Limitations

While diffusion-imaging studies of tinnitus aim to investigate changes in long-range connectivity, most findings to date (including those of the present study) are in fact quite localized, being based either on comparisons of diffusion measures in fairly small predefined regions of interest or a more global analysis yielding small, localized clusters of voxels showing significant effects. Drawing inferences about the integrity of long-range connections between fairly remote brain regions (as suggested by terms like “auditory-limbic connectivity”) from such localized results is difficult. Ideally, one would instead trace connections between auditory and limbic regions of interest and then assess the patency of these connections along their entire length (i.e., the approach taken by Crippa et al. [38]).

Unfortunately, the suitability of current fiber tracking methods for identifying the auditory and auditory-limbic connections of interest is questionable. Deterministic fiber tracking, which relies on the dominant diffusion direction as an indicator of fiber orientation in a voxel, cannot identify even major auditory pathways because they are crossed by more dominant orthogonal pathways into which the tracking algorithm gets diverted. Probabilistic fiber tracking is more flexible and able to trace nondominant fibers, but regarding current methods, its flexibility is also its biggest weakness, because it allows fiber samples to take quite implausible routes. Our own experience from attempting probabilistic fiber tracking in the present dataset (results not reported for reasons that will be laid out in the following) is well in line with the complications reported by Crippa et al. [38]. When tracking from one seed ROI to a target ROI, a significant number of fiber samples tend to take “shortcuts” through gray matter or even cerebrospinal fluid or they reach the target only after highly unlikely detours through remote brain areas. A common remedy for these problems is the use of “exclusion masks” defining areas where fibers are not allowed to go (such as the ventricles); if a fiber sample nevertheless reaches this area, it is aborted and does not figure into the results. In addition, Crippa et al. [38] also report manual removal of fiber samples leading to the cerebellum or motor cortex. Despite all this, fiber tracking in their study was successful for only 50 percent (or fewer, depending on the tract) of the participants. Crippa et al.'s [38] data, along with our own experience, indicate that probabilistic fiber tracking can accurately identify known auditory pathways (e.g., from the inferior colliculi via the medial geniculate nuclei to primary auditory cortex) if sufficiently constrained by the researcher based on prior knowledge. However, the fact that unconstrained fiber tracking can lead to quite implausible results casts significant doubt on the validity of plausible tracts that remain after effectively preventing implausible behavior. Furthermore, it also means that one cannot rely on the results of fiber tracking between ROIs whose actual anatomical connection is not known (e.g., on the basis of tracer studies in animals).

Moreover, even if tracking is successful and the identified tracts are plausible, making quantitative inferences about connectivity (whether interpreted as fiber density, myelination, or something else) from diffusion data is highly problematic for several reasons discussed elsewhere [68], which will be briefly summarized here. One approach for quantifying connectivity is to compute average FA along the identified tract (based on the reasoning that a loss of axons or a reduction in myelination of the dominant fiber bundle will reduce diffusion obstacles in the direction perpendicular to the bundle, thus reducing FA). Considering that, as outlined in the Introduction section, the vast majority of voxels contain more than one bundle of parallel fibers, it is obvious why this does not yield an interpretable measure of connectivity: FA is influenced by the crossing fibers as well, so that, for example, a reduction of FA can result not only from reductions of connectivity (e.g., fiber density or myelination) in the tract of interest, but also from an increase in connectivity along perpendicular, crossing tracts. Another approach related to probabilistic fiber tracking is to evaluate tracking success (e.g., assessing which proportion of streamlines sent out from a user-defined seed region reaches a user-defined target region); a related measure is to average tracking success from the seed region to each voxel along the tract of interest to obtain a measure of “connection probability.” However, these measures depend crucially on the tracking parameters (e.g., the maximum angle a streamline is allowed to bend from one voxel to the next without being aborted as biologically implausible), the length and curvature of the path to be tracked, the presence of crossing fibers or tract “branching” that might divert the streamline, and the overall signal-to-noise ratio in the images.

For these reasons, we think that diffusion imaging of tinnitus cannot yet confidently draw conclusions about “auditory system connectivity” or “auditory-limbic connectivity.” The results of studies performed to date are called into question by considerable technical difficulties (e.g., with intersubject alignment, fiber tracking, or controlling for potential confounding variables such as hearing loss, noise sensitivity, and depression), and inferences made from the results often go far beyond the data. Thus, while what we have so far may be valuable puzzle pieces, the emergence of the whole picture illustrating tinnitus-related changes in anatomical connectivity will likely have to wait until diffusion imaging and fiber tracking techniques are more advanced.

### 4.5. Future Directions

While it was the goal of this paper to alert the tinnitus research community to proceed with caution when using and interpreting the results of diffusion MRI, we by no means intend to discourage the use of the technique for tinnitus research. On the contrary, since tinnitus is increasingly thought of as a network problem and, due to its subjective nature, is most easily investigated in humans, thus requiring noninvasive methods, diffusion imaging may be one of the better tools available. Discontinuing its use altogether would be akin to throwing out the baby with the bathwater. We thus want to end this discussion with a few recommendations for future use of diffusion imaging in tinnitus research.

In general, as with any research method, it is important that those using diffusion imaging as a research tool understand the limitations of the technique. To this end, we refer the reader to Jones et al.'s “Do's and Do not's of diffusion MRI” [36], a recent review of diffusion imaging and related analysis methods that also makes recommendations for good practice. In addition to those recommendations, we would like to stress the importance of using intersubject alignment methods ensuring coregistration of corresponding WM tracts (as opposed to approaches that align based on the entire brain and apply large amounts of smoothing to “smooth over” intersubject differences in tract location). The TBSS approach described above has been successful in this respect, and a recent publication promises further improvements in this direction [69]. Also, to facilitate interpretation of the results, we recommend combining multiple diffusion measures, such as FA and MD.

Specifically for tinnitus research, it is crucial to assess tinnitus-related variables such as age, hearing-loss, noise sensitivity, depression, and anxiety and to take them into account when performing analyses. (While this has become common practice in fMRI studies of tinnitus, it seems to have been mostly neglected in diffusion imaging studies so far.) Since tinnitus-related changes in tissue microstructure are likely subtle, the small number of participants commonly enrolled in fMRI studies is probably insufficient for achieving the statistical power necessary to detect such small effects. (This criticism clearly applies to the present study, as evidenced by the fact that, for many of the reported findings, the opposite hemisphere showed clear trends in the same direction, which however failed to reach statistical significance.) It would thus be highly desirable if tinnitus researchers could agree upon collecting diffusion data in the same way and combining them into one big dataset. This could be achieved by polling tinnitus researchers interested in pursuing diffusion imaging at one of the upcoming tinnitus conferences and drafting a “consensus” paper, as has already been done for “tinnitus patient assessment and treatment outcome measurement” [49]. In addition to higher-powered cross-sectional studies, longitudinal studies, especially prospective ones that follow participants at high risk for developing tinnitus (e.g., active-duty military prior to deployment), could help answer the question of whether specific tinnitus-related changes identified in cross-sectional studies precede the tinnitus (i.e., there is a preexisting vulnerability) or develop after tinnitus onset (and thus likely reflect a consequence of the phantom percept rather than a cause).

## 5. Conclusions

Diffusion imaging is a noninvasive tool for assessing anatomical connectivity in the brain in vivo. As such, it has the potential to provide anatomical evidence for those tinnitus-related changes in auditory and auditory-limbic connectivity that have been proposed based on previous functional imaging and electrophysiological data. The few diffusion-imaging studies of tinnitus performed to date (including the present one) have somewhat divergent results whose interpretations are complicated by various technical difficulties. Despite this, there seems to be some converging evidence that hearing loss is associated with changes in diffusion measures thought to reflect decreases in anatomical connectivity of central auditory pathways that go beyond those that occur in the course of aging. In contrast, tinnitus is associated with changes that might reflect increases in auditory and auditory-limbic connectivity. Future research will have to confirm these findings and establish whether these connectivity increases are the cause of the tinnitus percept or rather a consequence of the tinnitus signal being continually propagated through the system. This enterprise will be greatly facilitated if researchers are willing to contribute to a shared dataset and agree upon and adhere to certain best practice approaches to analyzing and interpreting diffusion data.

## Figures and Tables

**Table 1:** ROIs.

ROI name (ROI actually refers to the WM tracts adjacent to the named structure)	Center of gravity (MNI coordinates)	Size (mm^3^)
Left inferior colliculus (lIC)	−6, −32, −15	27
Right inferior colliculus (rIC)	6, −32, −15	30
Left medial geniculate nucleus (lMGN)	−18, −24, −2	348
Right medial geniculate nucleus (rMGN)	18, −23, −2	349
Left auditory cortex (lACx)	−44, −29, 2	594
Right auditory cortex (rACx)	46, −26, 5	570
Left amygdala (lAM)	−28, −15, −10	70
Right amygdala (rAM)	28, −14, −10	42
Left nucleus accumbens (lNAc)	−15, 14, −1	48
Right nucleus accumbens (rNAc)	14, 14, −1	63
Left ventromedial prefrontal cortex (lvmPFC)	−19, 28, −9	183
Right ventromedial prefrontal cortex (rvmPFC)	20, 29, −9	199
Average WM skeleton (WM skeleton)		76259
